# Alterations in epidermal growth factor receptors 1 and 2 in esophageal squamous cell carcinomas

**DOI:** 10.1186/1471-2407-12-569

**Published:** 2012-12-04

**Authors:** Isabela Martins Gonzaga, Sheila Coelho Soares-Lima, Paulo Thiago Souza de Santos, Tania Cristina Moita Blanco, Bruno Souza Bianchi de Reis, Danielle Carvalho Quintella, Ivanir Martins de Oliveira, Paulo Antonio Silvestre de Faria, Cleber Dario Pinto Kruel, Nelson Adami Andreollo, Tatiana Almeida de Simão, Luis Felipe Ribeiro Pinto

**Affiliations:** 1 Programa de Carcinogênese Molecular, Instituto Nacional de Câncer, Coordenação de Pesquisa, Rua André Cavalcanti, 37 – 6º andar, Bairro de Fátima, Rio de Janeiro, Rio de Janeiro, CEP: 20231-050, Brazil; 2 Departamento de Bioquímica, Instituto de Biologia Roberto Alcantara Gomes, Universidade do Estado do Rio de Janeiro, Av. 28 de Setembro 87 fundos, Vila Isabel, Rio de Janeiro, CEP: 20551-013, Brazil; 3 Divisão de Patologia, Instituto Nacional de Câncer, Rua Cordeiro da Graça 156 Santo Cristo, Rio de Janeiro, Rio de Janeiro, CEP: 20.230-240, Brazil; 4 Hospital de Clínicas de Porto Alegre, PPG-Ciências Cirúrgicas-Famed, Universidade Federal do Rio Grande do Sul, Rua Ramiro Barcelos 2400 2o andar, Santana, Porto Alegre, Rio Grande do Sul, CEP: 90035-903, Brazil; 5 Departamento de Cirurgia e Gastrocentro, Faculdade de Ciências Médicas, Universidade Estadual de Campinas, Rua Alexandre Fleming 181, Barão Geraldo, Campinas, São Paulo, CEP: 13081-970, Brazil

**Keywords:** Esophageal cancer, EGFR, HER2, KRAS, BRAF, Target therapy

## Abstract

**Background:**

Esophageal squamous cell carcinoma (ESCC) shows a 5-year survival rate below 10%, demonstrating the urgency in improving its treatment. Alterations in epidermal growth factor receptors are closely related to malignancy transformation in a number of tumors and recent successful targeted therapies have been directed to these molecules. Therefore, in this study, we analyzed the expression of EGFR and HER2 and evaluated *EGFR* mutation profile as well as the presence of mutations in hotspots of *KRAS* and *BRAF* in ESCC patients.

**Methods:**

We performed RT-qPCR, immunohistochemistry and Fluorescent *in situ* hybridization to determine EGFR and HER2 expression in ESCC patients, and direct sequencing and PCR-RFLP for mutations and polymorphism analysis**.**

**Results:**

Our results showed an increased *EGFR* mRNA expression in tumors compared to surrounding tissue (p <0.05), with 11% of the cases presenting at least a four-fold difference between tumor and paired adjacent mucosa. EGFR protein overexpression was present only in 4% of the cases. The median expression of *HER2* mRNA was not different between tumors and adjacent mucosa. Still, 7% of the tumors presented at least a 25-fold higher expression of this gene when compared to its paired counterpart. Immunohistochemical analysis revealed that 21% of the tumors were positive for HER2 (scores 2+ and 3+), although only 3+ tumors presented amplification of this gene. Mutation analysis for *EGFR* (exons 18-21), *KRAS* (codons 12 and 13) and *BRAF* (V600E) showed no mutations in any of the hotspots of these genes in almost 100 patients analyzed. *EGFR* presented synonymous polymorphisms at codon 836 (C>T) in 2.1% of the patients, and at codon 787 (G>A) in 79.2% of the cases. This last polymorphism was also evaluated in 304 healthy controls, which presented a similar frequency (73.7%) in comparison with ESCC patients. The absence of mutations of *EGFR*, *KRAS* and *BRAF* as well as the overexpression of EGFR and HER2 in less than 10% of the patients suggest that this signaling pathway is altered in only a small proportion of patients with ESCC.

**Conclusion:**

HER receptors target therapies may have the potential to be effective in only a minor fraction of patients with ESCC.

## Background

Esophageal cancer (EC) is among the ten most incident tumors in the world, and esophageal squamous cell carcinoma (ESCC) is the most frequent type of EC. In addition to its high incidence, ESCC ranks fifth in cancer mortality in men and eighth in women. ESCC mortality and incidence rates are similar, with the 5 year overall survival rate being below 15% [1,2]. The poor prognosis of ESCC patients results from late stage diagnosis and the poor efficacy of treatment, with systemic chemotherapy having mainly a palliative role [3]. Although a number of cytotoxic drugs have been used to treat ESCC patients, overall survival rates have not improved [4].

Therefore, the development of new therapy modalities, particularly targeted therapies based on the knowledge of the biology and genetics of the disease may offer a potential for improving treatment response and life quality for ESCC patients [5]. Drugs targeting the human epidermal growth factor receptors (HER) may act in two manners: as tyrosine kinase activity inhibitors (TKIs) or as receptor blocking monoclonal antibodies (mAbs) [6]. A number of these drugs, such as gefitinib used to treat non-small cell lung cancer, cetuximab used to treat patients diagnosed with advanced colorectal cancer, and particularly trastuzumab used to treat breast cancer patients, have shown substantial improvement in tumor response when compared with conventional chemotherapy [7-9].

Among HER family members, EGFR (HER1) and HER2 are the most commonly altered receptors in human malignancies [10]. These receptors are mainly involved in cell proliferation and survival through activation of PI3K-Akt [11], STAT3 [12], and Ras-Raf-MAPK signaling pathway, with the latter described as the main pathway activated by EGFR [13]. The most common EGFR alterations found in tumors are mRNA and protein overexpression, often associated with gene amplification, followed by mutations in specific hotspots located in the region that encodes the tyrosine-kinase domain of the receptor [14]. The increased expression of EGFR is mainly found in head and neck cancers, in which 70-90% of the cases show this profile [15]. Complementary, *EGFR* mutations were firstly reported in lung cancer patients who had greater response to treatment with EGFR tyrosine kinase inhibitors. These mutations are generally found in the exons 18-21 of the gene and are more prevalent in Asian non-smoker women with lung adenocarcinoma [16]. The role of HER2 in tumorigenesis is a consequence of abnormally increased protein expression, as a result of gene amplification. This phenomenon is observed in more than 25% of breast cancer patients and more recently in about 15-25% of gastric cancer patients [17,18].

In addition to the alterations in HER receptors, mutations in genes involved in the signaling pathways activated by these receptors are also correlated with the carcinogenesis process and failure of therapeutic response to HER inhibitors [14]. For instance, colorectal cancer patients who present mutations in *KRAS* or *BRAF* do not respond to panitumumab, a monoclonal antibody against EGFR, recently approved by FDA as a monotherapy against metastatic colorectal carcinoma [19].

Since EGFR and HER2 alterations may predict a successful response to HER target specific therapy, and ESCC has a very poor prognosis with currently available treatments, it is essential to analyze possible alterations of these receptors in ESCC, to evaluate the potential of use of anti-HER therapy to treat ESCC patients.

## Methods

### Samples

Two-hundred and forty one patients with a confirmed histologically diagnosis of ESCC who had not undergone chemo or radiotherapy were recruited between 1997 and 2010 from four hospitals in Brazil: Hospital Universitário Pedro Ernesto (HUPE-UERJ, Rio de Janeiro), Instituto Nacional do Câncer (INCA, Rio de Janeiro), Hospital de Clínicas (HCPA-UFRGS, Porto Alegre), and Hospital de Clínicas-Gastrocentro (HC-UNICAMP, Campinas). Tumor and adjacent mucosa were obtained either as formalin fixed paraffin embedded (FFPE), or fresh snap frozen tissue. Patients’ information was collected either from their medical records, or from a standardized questionnaire. In addition to patients, 304 subjects without cancer were included in the study (control group), from whom 5 mL of peripheral blood were collected. The controls also answered the standardized questionnaire and all patients signed a consent form. The project was approved by the Ethic Committees of all institutions involved.

#### DNA and RNA isolation

The DNA isolation from frozen samples was performed according to Sambrook and colleagues [20], while DNA isolation from FFPE samples was carried out using the QIAamp DNA FFPE Tissue kit (QIAGEN®, Germany). DNA was also isolated from blood lymphocyte (control group) using the proteinase K/sodium dodecyl sulfate digestion as described by Miller and colleagues [21]. Finally, total RNA was extracted from tissues using the TRIzol® (Invitrogen, USA) reagent following the protocol described by the manufacturer. All RNA samples were quantified by spectrophotometry and their integrity was evaluated by formaldehyde-agarose gel electrophoresis. The quality of the RNA samples was determined by the ratio of the 28S, 18S and 5.8S ribosomal RNA bands.

#### PCR and direct sequencing

In order to assess the viability of DNA extracted from FFPE samples, amplification of *β-actin* was performed. Amplification of *EGFR* (exons 18- 21) [22], *KRAS* (exon 2), *BRAF* (exon 15) and *β-actin* was done according to the following protocol: 1X PCR buffer (Invitrogen, USA), 3 mM MgCl_2_ (Invitrogen, USA), 0.2 mM dNTPs, 0.5 U of *GoTaq Polymerase* (Promega, USA), 3 pmol of each primer up to 25 μL. For amplification, the DNA was first denatured for 5 min at 94°C and followed by 40 PCR cycles consisting of three steps: denaturation for 30 seconds at 92°C, annealing for 1 minute at specific primer annealing temperature and extension for 1 minute at 72°C. To assess *β-actin* amplification we used 100 ng of genomic DNA, while for *KRAS* and *BRAF* analysis we used 300 ng of DNA from FFPE and 100 ng of DNA from frozen samples. All oligonucleotides used are summarized in Table 1. PCR products were then purified with the PureLink™ Genomic DNA Purification kit according to the manufacturer’s protocol (Invitrogen, USA). Sequencing reactions contained 2 μL of purified PCR product, 40 ng of primer (sense or anti sense) and 2 μL of the kit (ET Dye Terminator Cycle Sequencing Kit - GE® Healthcare, UK) and were analyzed on a MegaBACE 1000 automatic sequencer (GE Healthcare, UK).

**Table 1:** Conditions of PCR reactions: oligonucleotide sequences, annealing temperatures, number of cycles of the reactions, and amplicon size

**Genes**		**Oligonucleotide sequences (5²-3²)**	**AT**^**a**^	**Cycles**	**Amplicon size (bp)**	**Reference**
*EGFR* – exon 18	S	F: CAAATGAGCTGGCAAGTGCCGTGTC	56°C	35	400	[22]
	AS	R: GAGTTTCCCAAACACTCAGTGAAAC				
*EGFR* – exon 19	S	F: GCAATATCAGCCTTAGGTGCGGCTC	58°C	35	372	[22]
	AS	R: CATAGAAAGTGAACATTTAGGATGTG				
*EGFR* – exon 20	S	F: CCATGAGTACGTATTTTGAAACTC	54°C	35	408	[22]
	AS	R: CATATCCCCATGGCAAACTCTTGC				
*EGFR* – exon 21	S	F: TAACGTTCGCCAGCCATAAGTCC	58°C	35	414	[22]
	AS	R: GCTGCGAGCTCACCCAGAATGTCTGG				
*β-actin*	S	F: GATGAGATTGGCATGGCTTT	55°C	40	100	b
	AS	R: CACCTTCACCCGTTCCAGTTT				
*BRAF*	S	F: CCTTTACTTACTACACCTCAGATA	54°C	40	189	b
	AS	R: AATCAGTGGAAAAATAGCCT				
*KRAS*	S	F: TGATAGTGTATTAACCTTATGTGTGAC	54°C	40	170	b
	AS	R: TCTATTGTTGGATCATATTCGTC				
*GAPDH*	S	F: CAACAGCCTCAAGATCATCAGCAA	60°C	40	123	b
	AS	R: AGTGATGGCATGGACTGTGGTCAT				
*EGFR*	S	F: TAACAAGCTCACGCAGTTGG	60°C	40	178	b
	AS	R: GTTGAGGGCAATGAGGACAT				
*HER2*	S	F: CTCCTGTGTGGACCTGGATGAC	60°C	40	143	b
	AS	R: GCTGCCGTCGCTTGATGA				

ª Annealing Temperature.

^b^ Designed by authors.

#### RT-qPCR

In order to synthetize cDNA, two to four micrograms of total RNA were used in reverse transcription (RT) reactions as previously described [23]. Equal amounts of RNA samples from the same patient (tumor and adjacent mucosa) were used in separate RT reactions.

For the individual evaluation of *EGFR, HER2* and *GAPDH* expression, one pair of primers spanning intron-exon junctions were designed and are described in Table 1. The PCR was performed in the thermocycler Chromo 4 (MJ Research®). Each reaction consisted of 7.5 μL of Faster EvaGreen 2X Master Mix® (Biotium, CA, USA), 10 pmols of oligonucleotide, 2 μL of cDNA (diluted 10X) and sterile deionized water to complete the final volume of 15 μL. The amplification reaction was performed as follows: five minutes of pre-denaturation at 95°C, followed by 40 cycles of denaturation for 15 seconds at 95°C and an annealing and extension step for 1 minute at 60°C. After the reaction, *EGFR* and *HER2* mRNA expression was normalized by the expression of *GAPDH*. The mRNA relative quantitation was done using the ΔCt method. The parameter Ct (threshold) was defined as the number of cycles in which the fluorescence exceeded the previously set threshold. The difference (ΔCt) between the average (three experiments) of the gene of interest (*EGFR* or *HER2*) and the housekeeping gene (*GAPDH*) was calculated using the software Microsoft Excel.

#### PCR-RFLP

The *EGFR* gene polymorphism was determined using the PCR-RFLP method. New primers were designed to proceed restriction endonuclease reaction (EGFR Sense: 5^′^CATGAGTACGTATTTTGAAACTC3^′^; and Anti-sense: 5^′^CACACACCAGTTGAGCAGGTA3^′^) and the PCR reaction was performed as follows: 25 ng of genomic DNA, 1X PCR buffer (Invitrogen, USA), 3 mM MgCl_2_ (Invitrogen, USA), 0.2 mM dNTPs, 0.5 U of *GoTaq Polymerase* (Promega, USA), 3 pmol of each primer up to 25 μL. For amplification, the DNA was first denatured for 5 min at 94°C and followed by 35 cycles consisting of three steps: denaturation for 30 seconds at 92°C, annealing for 1 minute at 58°C annealing temperature and extension for 1 minute at 72°C. Two microliters of the PCR product (410 bp) were incubated with 2.5 U of BsgI (New England Biolabs®) for 18 hours at 37°C, and the resulting fragments were visualized on a 2.5% agarose gel stained with SYBR® Safe (Invitrogen®). The genotypes were classified as wild type homozygous (95 and 201 bp), heterozygous (95, 201 and 296 bp) and variant homozygous (296 pb).

#### Immunohistochemistry (IHC)

Immunohistochemistry was performed on paraffin sections of 69 ESCC cases. For antigen retrieval, sections were incubated in a pressure cooker while submerged in a citrate buffer solution, pH 6.0, for 3 min at 121°C. Sections were incubated with the primary antibody against EGFR (Code 4267 - Cell Signaling®; diluted 1:300 in diluent solution) [24] and HER2 (Code-A048529 1 -® Dako; diluted 1:4000 in diluent solution) [25] overnight at 4°C. Sections were then washed and covered with biotinylated secondary antibody for 30 min at room temperature followed by incubation in streptavidin–peroxidase solution for 30 min. The detection system was a Detection Novolink Polymer Systems (Leica Biosystems®), using diaminobenzidine (DAB) as substrate. Sections were counterstained with Harris’ hematoxylin. FFPE lung and breast cancer tissue served as positive controls of EGFR and HER2, respectively. For a negative control, the primary antibody was replaced with the antibody diluent solution.

The staining score evaluation was performed by two independent pathologists. For HER2 scores, we used the HercepTest™ (Dako®) indicated to assess HER2 staining in breast cancer, with a similar cut-point of 10% of positive tumor cells used to consider positive staining for HER2. To evaluate EGFR staining score, we used the method described by Pirker and colleagues [26] as follows:

$$\left(1\text{x}\right)+\left(2\text{y}\right)+\left(3\text{z}\right)\;\geq \;200\;\left(\text{Positive}\right)$$

$$\left(1\text{x}\right)+\left(2\text{y}\right)+\left(3\text{z}\right)\;\text{<}\;200\;\left(\text{Negative}\right)$$

Where x is the percentage of tumor cells with 1+ score (weak staining), y is the percentage of tumor cells with 2+ score (moderate staining) and z is the percentage of tumor cells with 3+ score (strong staining).

#### Fluorescent *in situ* hybridization (FISH)

The cases classified as HER2 positive in immunohistochemistry analysis (2+ and 3+ scores) were subjected to gene amplification analysis by FISH using the HER2 FISH pharmDx™ kit (Dako®). Tissue sections (3 μm) were incubated for 30 minutes in a solution of 0.2 N HCl at room temperature. Then, they were immersed in citrate buffer pH 6.0 for 30 minutes at 98°C and followed the manufacturer’s protocol. To evaluate *HER2* amplification we counted the red (*HER2*) and green signals (Centromere 17 - CEN17) in twenty nuclei of each tumor. If *HER2*/CEN17 ≤ 1.8, the sample is classified as non-amplified; 1.8 <*HER2*/CEN17 ≤ 2.2, as indeterminate status; and if *HER2*/CEN17> 2.2, the sample was classified as amplified. The adjacent normal tissues were used as internal controls of the reaction.

#### Statistical analysis

Allele frequencies of *EGFR* were calculated and tested for Hardy-Weinberg equilibrium within cases and controls. To determine if there were differences in mRNA expression of *EGFR* and *HER2* in tumor when compared to paired adjacent mucosa we used Wilcoxon signed-rank test. Outliers were assessed by Grubbs test. All statistical analysis was performed with GraphPad Prism 5 (GraphPad Software, USA).

The total number of patients (241) was divided into smaller groups according to the analysis performed, due to heterogeneity in sample quality. The RT-qPCR and sequencing analyses had to additionally rely on a number of frozen tumors to reach acceptable statistical power [27].

## Results

### Patients and tumors characteristics

The characteristics of the patients are summarized in Table 2. The median age of patients was 58 years, ranging from 34 to 88 years, with most of the patients being male (64%), alcohol drinkers (58%) and smokers (65%), with a median tobacco consumption above 30 packs/year. The tumors were located most often in the middle third of the esophagus, with a higher prevalence of T3 and T4 classification.

**Table 2:** Characteristics of the individuals included in this study (% of the total of patients)

	**Controls**	**Patients**
**Samples, n(%)**		
FFPE samples	-	98 (41)
Frozen Samples	-	143 (59)
Blood samples	304 (100)	-
Total	304 (100)	241 (100)
**Gender, n(%)**		
Male	140 (46)	154 (64)
Female	163 (54)	50 (21)
**Median Age (years)**	54	58
Range	19-89	34-88
**Alcohol Consumption, n(%)**		
Never drink	214 (70)	30 (12)
Current Drinker	83 (27)	139 (58)
**Tobacco Consumption, n(%)**		
Never smoke	133 (44)	18 (7)
Current Smoker	164 (54)	157 (65)
**Packs/Year Index, n(%)**		
≤ 30	92 (69)	63 (26)
> 30	24 (18)	88 (37)
**T (TNM), n(%)**		
Ti	-	1 (0,4)
T1	-	10 (4)
T2	-	16 (7)
T3	-	61 (25)
T4	-	34 (14)

*Number of patients may vary due to missing data.

### EGFR alterations in ESCC

#### *EGFR*, *KRAS* and *BRAF* mutations

Initially we analyzed potential alterations in exons 18 to 21 of *EGFR*, and found no mutations among the 135 samples studied. However, a synonymous polymorphism in exon 20 (Q787Q - G2607A; ID: rs1050171) was identified in 107 patients (79%). The genotypes were distributed as follows: 28 (21%) wild-type homozygous (GG), 72 (53%) heterozygous (AG) and 35 (26%) variant homozygous (AA). The genotypic frequencies were in Hardy-Weinberg equilibrium (p> 0.05). In addition, another synonymous polymorphism was found in exon 21 (R836R - C2754T; ID: rs17290559) in three patients (2%), all heterozygous.

Due to the high frequency of the G2607A polymorphism in ESCC patients, we decided to investigate whether this variant confers a risk for esophageal cancer development in a case-control study. With this purpose, the presence of this polymorphism was assessed by PCR-RFLP in a group of 304 individuals without cancer. Out of the 304 subjects, 80 (26%) were wild-type, 138 (45%) were heterozygous and 86 (28%) were variant homozygous. The genotypic frequencies were in Hardy-Weinberg equilibrium (p> 0.05) and there was no association between the presence of the polymorphism and ESCC (p> 0.05) (Table 3).

**Table 3:** **Genotype frequencies of *EGFR *polymorphism G2607A in ESCC patients and control group**

**G2361A *EGFR *polymorphism**	**Patients**	**Controls**
Wild-type homozygous (GG)	28 (21%)	80 (26%)
Heterozygous (GA)	72 (53%)	138 (45%)
Variant homozygous (AA)	35 (26%)	86 (28%)
Total	135 (100%)	304 (100%)

A total of 91 samples were investigated for the presence of potential mutations in *KRAS* (codons 12 and 13) and *BRAF* (V600E), with none of them being positive.

#### EGFR expression

**Figure 1:**
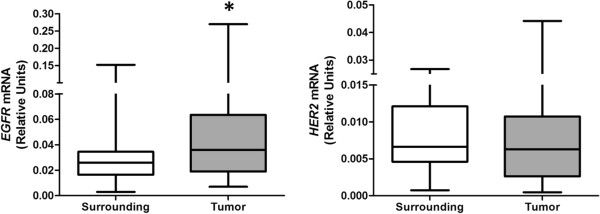
**Analysis of mRNA expression of *EGFR *and *HER2 *in ESCC patients:(A)Comparison of *EGFR *mRNA expression between tumor and normal adjacent mucosa of 37 ESCC patients.** (**B**) Comparison of *HER2* mRNA expression between tumor and normal adjacent mucosa of 30 ESCC patients.

The mRNA expression profile of *EGFR* was analyzed in 37 matched samples (tumor and adjacent tissue) with a higher median *EGFR* expression in tumors in comparison with surrounding mucosa (p <0.05) (Figure 1A). The paired sample analysis revealed that 16 (43%) tumors showed at least a 1.5-fold higher expression of *EGFR* when compared with the adjacent mucosa. Among these, 25% (11% of all samples) showed an overexpression above 4-fold (ranging from 4.2- to 9.7-fold), and these were confirmed as outliers (p <0.05).

**Figure 2:**
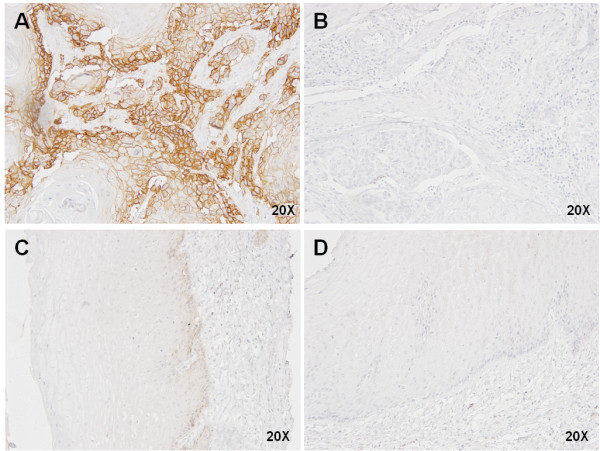
**Expression of EGFR in ESCC by immunohistochemistry.** (**A**) Representative figure of a EGFR positive staining case in ESCC, and (**B**) its corresponding negative control. (**C**) Representative figure of a EGFR negative case in ESCC, and (**D**) its corresponding negative control.

Next, we evaluated EGFR protein expression by immunohistochemistry in 69 ESCC samples. Sixty-six tumors (96%) were classified as negative for EGFR staining, while only three (4%) showed EGFR positive staining in the tumor area (Figure 2). The staining was localized mainly in the cell membrane with a weaker staining in cytoplasm. All of the three positive cases presented EGFR staining in the entire tumor. However, one case showed a heterogeneous staining, while the other two cases presented a homogeneous EGFR staining. The adjacent normal tissue showed a weak homogeneous staining predominantly localized in the basal layer.

The tumors that presented the highest EGFR expression were not correlated with any of the clinicopathological parameters analyzed in this study.

### HER2 alterations in ESCC

#### HER2 expression and amplification

The evaluation of *HER2* mRNA levels included 30 matched samples (tumor and adjacent tissue). There was no difference in the median expression of *HER2* expression in tumors in comparison with the surrounding tissue (p> 0.05) (Figure 1B). However, two samples (7%) showed *HER2* overexpression higher than 25-fold (25.2- and 37.8-fold) in tumor tissue when compared to its matched adjacent mucosa, which were confirmed as outliers (p <0.05).

**Figure 3:**
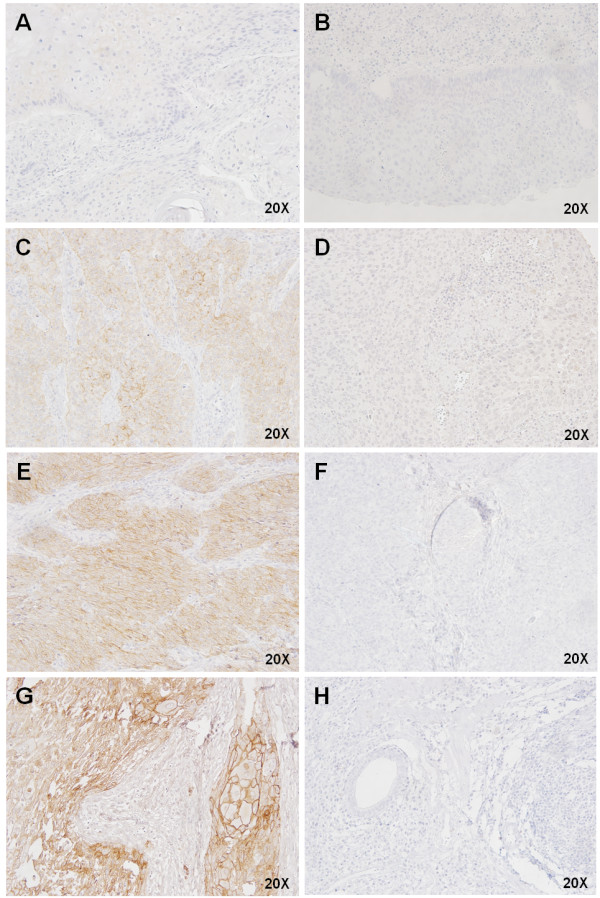
**Expression of HER2 in ESCC by immunohistochemistry.** (**A**) Representative image of HER2 negative score in ESCC, and (**B**) its corresponding negative control. (**C**) Representative image of HER 2 Score 1+ in ESCC, and (**D**) its corresponding negative control. (**E**) Representative image of HER 2 Score 2+ in ESCC, and (**F**) its corresponding negative control. (**G**) Representative image of HER 2 Score 3+ in ESCC, and (H) its corresponding negative control.

HER2 protein expression was also analyzed by immunohistochemistry in 68 ESCC samples. A total of 39 tumors (57%) were negative for HER2 staining, 14 (21%) were classified as score 1+, 12 (18%) were scored 2+ and only 3 (4%) were scored 3+. Thus, 53 ESCC patients (78%) were negative for HER2 expression (negative and 1+ scores) and 15 cases (22%) were initially considered positive (2+ and 3+ scores) (Figure 3). A similar expression profile to that seen for EGFR was observed with HER2 positive staining. The adjacent normal tissue also presented a weak homogeneous staining in the basal layer.

**Figure 4:**
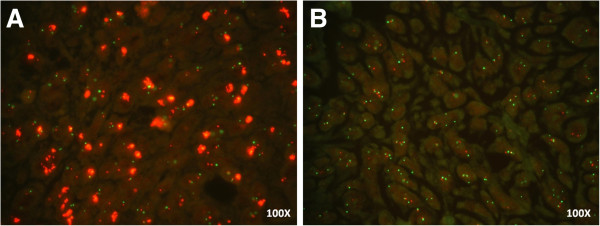
**HER2 gene amplification by FISH.** (**A**) Representative image of a positive case for *HER2* amplification in ESCC. (**B**) Representative image of a negative case for *HER2* amplification in ESCC.

Among the 15 cases (22%) initially classified as HER2 positive by IHC, we were able to analyze gene amplification by FISH on 11 (nine 2+ and two 3+) due to low material availability in 4 samples. Amplification of *HER2* was confirmed in the two cases classified as score 3+ by IHC. One sample presented a heterogeneous labeling along the tumor field with some areas presenting gene amplification and areas with normal signal (non-amplified). The other sample exhibited a homogeneous signal, in which the entire tumor extension showed *HER2* amplification (Figure 4A). Score 2+ samples showed no gene amplification (Figure 4B).

The tumors that presented the highest HER2 expression were not correlated with any of the clinicopathological parameters analyzed in this study.

## Discussion

The present study revealed that ESCC of Brazilian patients, who largelly present typical western characteristics, do not present mutations in hot spots of *EGFR* (exons 18-21), *K-RAS* (codons 12 and 13) and *BRAF* (V600E), and only a minor proportion (4%) present overexpression of EGFR or HER2. These results indicate that common alterations in EGFR and HER2 receptors and in the Ras-Raf-MAPK signalling pathway, observed in many other epithelial tumors, are rare in ESCC from Brazilian patients.

EGFR alterations in cancer can be divided mostly in two categories: mutations in exons 18-21, which encode the tyrosine kinase portion of the receptor, and gene and protein overexpression. *EGFR* mutations are mostly observed in lung tumors, and curiously they are more prevalent in Asian women diagnosed with adenocarcinoma who never smoked [16]. The most frequent *EGFR* mutations are deletions in exon 19 and a point mutation in codon 858 of exon 21, known as L858R (T2573G; ID: rs121434568) [16]. Patients who carry these mutations in *EGFR* tend to have a better response to gefitinib, an EGFR-TKI, whereas patients with the wild-type genotype show a better response to conventional chemotherapy [7]. This could be explained by the fact that the mutated receptor possess a greater affinity to the drug in comparison with ATP, and therefore cannot initiate the phosphorylation cascade downstream through the signaling pathways that lead to proliferation and cell survival. However, about 50% of lung cancer patients treated with EGFR-TKI acquire a secondary mutation that confers drug resistance, the T790M (C2369G; ID: rs121434569), located in exon 21 of the gene, which reduces the affinity of the ATP-binding site for the drug [15]. In addition to lung cancer, other tumors present low frequencies of *EGFR* mutations, like head and neck cancers, with no more than 7% of the patients carrying these alterations [28]. Our results showed no mutations in exons 18 to 21 of *EGFR* in 135 ESCC patients. So far, few studies were published that analyzed mutations in EGFR in ESCCs [29-31]. Among these, only one report found mutations in this gene (in 14% of tumors). However this study was carried out with Chinese patients, who usually present a different set of etiological factors when compared to western patients. Furthermore, the authors used the Scorpions Amplification Refractory Mutation System, a non-conventional methodology for the identification of mutations [31]. Our study also identified two synonymous polymorphisms: one at codon 787, in exon 20, with a G>A transition, found in more than 79% of the patients, without any significant difference to controls, and another at codon 836, in exon 21, with a C>T transition in only 2% of the patients.

It is estimated that 33-50% of epidermal tumors present overexpression of EGFR [14], being observed in more than 90% of head and neck tumors [15]. In addition to protein overexpression, around 10-17% of the head and neck tumors present *EGFR* gene amplification, as shown by FISH analysis [28]. In 2006, the FDA approved the use of cetuximab, a chimeric anti-EGFR mAb, for the treatment of patients with head and neck tumors presenting overexpression of this protein. The use of cetuximab was approved for the first time in 2004 for the treatment of colorectal cancer, which has high response rates to this drug (about 47% of the patients) [8], although there is no concordance in the literature about the role of EGFR expression as a biomarker for response to this targeted therapy [32-34]. More recently, Panitumumab, a humanized anti-EGFR mAb, was also approved to colorectal cancer treatment, with good results in therapeutic efficacy [35]. However, several reports showed that mutations in genes involved in the Ras-Raf-MAPK pathway, like *KRAS* and *BRAF*, are important biomarkers for colorectal tumor patient response to anti-EGFR mAbs. These mutations turn these proteins constitutively activated, resulting in a receptor-independent activation of the pathway, what culminates in the resistance to treatment with anti-EGFR mAbs [36]. The most frequent mutations observed in colorectal cancer patients are found at codons 12 and 13 of *KRAS*, in approximately 35% of the patients, and the V600E mutation of *BRAF*, found in about 15% of the cases [19,34]. Head and neck tumors present mutations in *KRAS* and *BRAF,* but in very low frequencies, with 6% of the patients carrying a mutation in *KRAS* and 3% in *BRAF*[37]. In our study, 11% of ESCC tumors presented elevated *EGFR* mRNA levels in comparison with the normal adjacent mucosa, while only 4% showed protein overexpression. Previous studies analyzing EGFR expression in ESCC showed protein overexpression in more than 40% of ESCC patients, with 15% of cases presenting gene amplification [30,38]. This difference may be explained by the different methodologies used to score EGFR staining by IHC. In this study we evaluated EGFR staining score by the method reported by Pierker *et al.*[26], where a sample with weak staining is not considered positive for EGFR expression. In the other studies [30,38], the scoring method adopted was less stringent. Nevertheless, differences among the populations that took part in our and in the other studies may also explain this difference.

We found no alterations in hotspots of *KRAS* and *BRAF* in ESCC patients. This data is in accordance with the study developed by Hollstein and colleagues, who previously described the absence of mutations in *KRAS* in ESCC of patients from Normandy (France) and Uruguay [39], while no study had investigated *BRAF* mutations in ESCC so far. Therefore, our results both on *EGFR* hot-spot mutations and expression suggest that the EGFR-Ras-Raf-MAPK pathway is not associated with esophageal carcinogenesis.

HER2 overexpression, as a consequence of gene amplification, was initially seen to be present in around 25% of breast cancer patients, and more recently in a similar percentage of stomach and esophagogastric junction tumors [40]. These findings became even more relevant with the possibility to use a HER2-specific antibody, trastuzumab, to treat these patients [41]. Breast cancer patients, who present HER2 overexpression and gene amplification, and are treated with trastuzumab present a response rate of 62%, that is substantially higher when compared with 32% achieved with conventional chemotherapy [9]. Our work demonstrated that 7% of the ESCC tumors show high *HER2* mRNA levels compared to the adjacent tissue, whereas 22% showed protein overexpression. Gene amplification was confirmed in 4% of the cases by FISH, a frequency comparable to that of increased mRNA levels. Some studies focused on ESCC already described a 3-fold higher frequency of patients with score 2+ for HER2 in comparison with those with score 3+. Besides, those reports also showed that every sample classified as score 3+ presented *HER2* amplification, similarly to our findings [42,43]. Interestingly, the frequency of cases with high *HER2* mRNA expression and gene amplification is much lower than those with protein overexpression, which could be explained by HER2 biology. It has been described previously that dimmers containing HER2 generally tend to remain longer in the plasma membrane and are not targeted for proteolytic degradation, returning to the membrane in a process called recycling [44]. This phenomenon could explain why cases scored as 2+, considered as protein overexpression, do not show gene amplification.

A limitation of this study was that although we initially had 241 tumor samples, these were divided into smaller groups according to the different assays performed, due to the heterogeneity in sample amount and quality. Although this solution may have generated results with a limited number of samples in some of the analyses, a sufficient statistical power was reached in all cases [27]. Therefore, we may suggest that HER-activated pathway does not play a predominant role in esophageal carcinogenesis in the vast majority of cases. Furthermore, the absence of any *EGFR*, *KRAS* and *BRAF* mutations as well as a frequency of HER overexpression of less than 10% may also suggest that these modifications could be lethal to esophageal cells during transformation. In accordance with this speculation, Kim and colleges showed that EGFR-induced human esophageal tumor presents a strong TUNEL staining [45], what suggests that EGFR overexpression tends to induce apoptosis pathways in esophageal cells. However, other *in vitro* studies are still necessary to confirm this hypothesis.

## Conclusion

This study shows that most ESCC patients do not have the molecular profile for anti-HER targeted therapy. Thus, other markers should be investigated in the pursuit of new treatments that could increase survival and life quality of these patients.

## Abbreviations

Ct: Cycle threshold; DAB: Diaminobenzidine; EC: Esophageal cancer; ESCC: Esophageal squamous cell carcinoma; FFPE: Formalin fixed paraffin embedded tissue; FISH: Fluorescent in situ hybridization; IHC: Immunohistochemistry; mAbs: Monoclonal antibodies; mRNA: Messenger RNA; PCR: Polymerase chain reaction; PCR-RFLP: Polymerase chain reaction followed by restriction fragment length polymorphism; RT: Reverse transcription; RT-qPCR: Reverse transcription followed by a quantitative polymerase chain reaction; TKIs: Tyrosine kinase activity inhibitors.

## Competing interests

The authors declare no competing interests.

## Authors' contributions

IMG, SCSL and TAS performed the experiments. LFRP coordinated the project. IMG, SCSL and LFRP wrote the manuscript. TCMB, IMO and BB evaluated sample quality control and performed the pathological analyses. DCQ performed the FISH analyses. PASF, CDPK and NAA participated in the collection of samples and study design. PTSS performed the statistical analyses and participated in the study design. All authors discussed the results and manuscript text. All authors read and approved the final manuscript.

## Pre-publication history

The pre-publication history for this paper can be accessed here:

http://www.biomedcentral.com/1471-2407/12/569/prepub
